# Supplementary material for: Ubiquitin ligase CHFR mediated degradation of VE-cadherin through ubiquitylation disrupts endothelial adherens junctions
Source: Nat Commun. 2023 Oct 18;14:6582. doi: 10.1038/s41467-023-42225-2 (PMC10584835; doi:10.1038/s41467-023-42225-2)
Supplement: Supplementary file 1 — Supplementary Information [file 41467_2023_42225_MOESM1_ESM.pdf]

Supplementary Information

"Ubiquitin Ligase CHFR Mediated Degradation of VE-cadherin through Ubiquitylation Disrupts Endothelial Adherens Junctions" (Tiruppathi et al.)

Supplementary Figures:

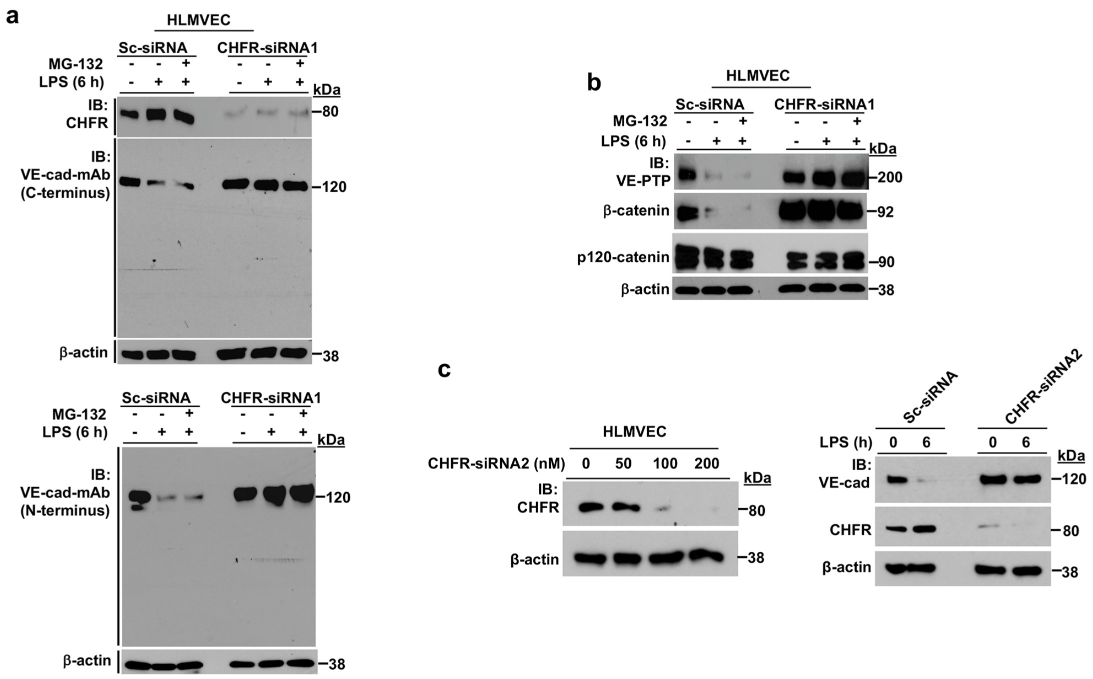

Supplementary Fig. 1. CHFR controls VE-cadherin expression at endothelial AJs. a, HLMVECs were transfected with 100 nM of either scrambled-siRNA (Sc-siRNA) or CHFR-siRNA1. At 72 h after transfection, cells pretreated with or without MG132 (10 μM) for 4 h were challenged with LPS (5 μg/ml) for 6 h and then cell lysates were used for IB analysis. *Top panel*, cell lysates were blotted with antibodies against CHFR and C-terminus of VE-cadherin. *Bottom panel*, cell lysates were blotted with antibody against N-terminus of VE-cadherin. **b**, HLMVEC lysates as in **a** were used for IB analysis to determine VE-PTP, p120-catenin, and β-catenin. **c**, *Left panel*, HLMVEC were transfected varying concentrations of CHFR-siRNA2 (from Dharmacon). At 72 h after transfection, cells were used for IB analysis. *Right panel,* HLMVECs were transfected with 100 nM of either scrambled-siRNA (sc-siRNA) or CHFR-siRNA2. At 72 h after transfection, cells challenged with LPS (5 μg/ml) for 6 h and then cell lysates were used for IB analysis. (**a-c,** n =2 independent experiments).

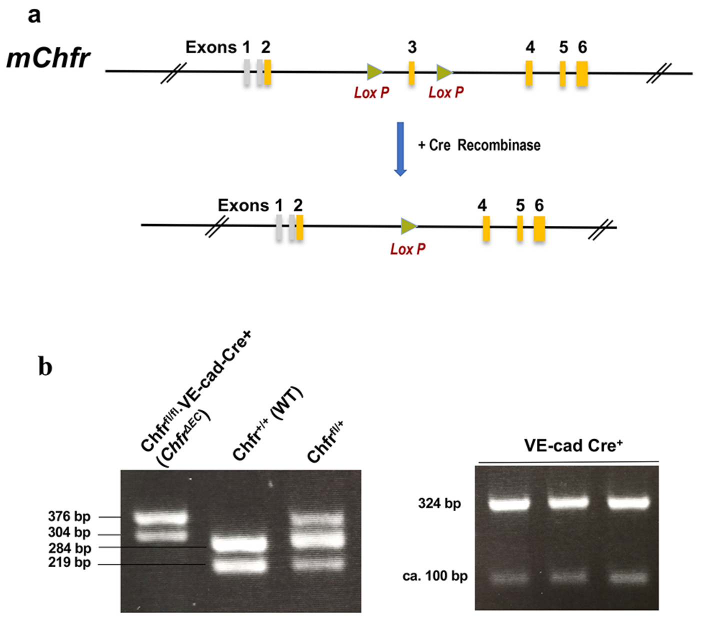

Supplementary Fig. 2. Generation of EC-restricted *Chfr* knockout (*Chfr*$^{\Delta EC}$) mice. a, Schematic representation of *mChfr* floxed genomic locus. In the scheme exons 1-6 are represented. Exon 3 of *mChfr* is flanked by loxP sites and the *mChfr* gene expression remains unaltered. *Cre* recombinase mediated deletion of floxed sites leads to a non-functional *mChfr* gene. **b**, **Genotyping of *Chfr*$^{fl/fl\text{-}VE\text{-}cad\text{-}Cre+}$ (*Chfr*$^{\Delta EC}$) mice.** Two *Chfr* primer pairs (*Chfr*$^{fl/fl}$; 304/376 bp; 5' loxp, forward: 5'-TCC TGG CCT TGG CTC ATT CC-3'; reverse: 5'- TCC TGC CAG AGC ATG AAG CA-3'; floxed band (304 bp) and wild-type (WT) band (219 bp); 3' loxp, forward: 5'-CTT TTG AAG GGA CAC ATT GCT TCA G-3'; reverse: 5'- AAC TTC TTC CCA GCT TCT ATG CCT T-3'; floxed band (376 bp) and WT band (284 bp). Two VE-cad-Cre primer pairs: forward 5'-GCG GTC TGG CAG TAA AAA CTA TC-3'; reverse 5'- GTG AAA CAG CAT TGC TGT CAC TT-3'; ≈ 100 bp; internal positive control: forward 5'-CTA GGC CAC AGA ATT GAA AGA TCT-3'; reverse 5'- GTA GGT GGA AAT TCT AGC ATC ATC C -3'; (324 bp) are required for verifying the mutation.

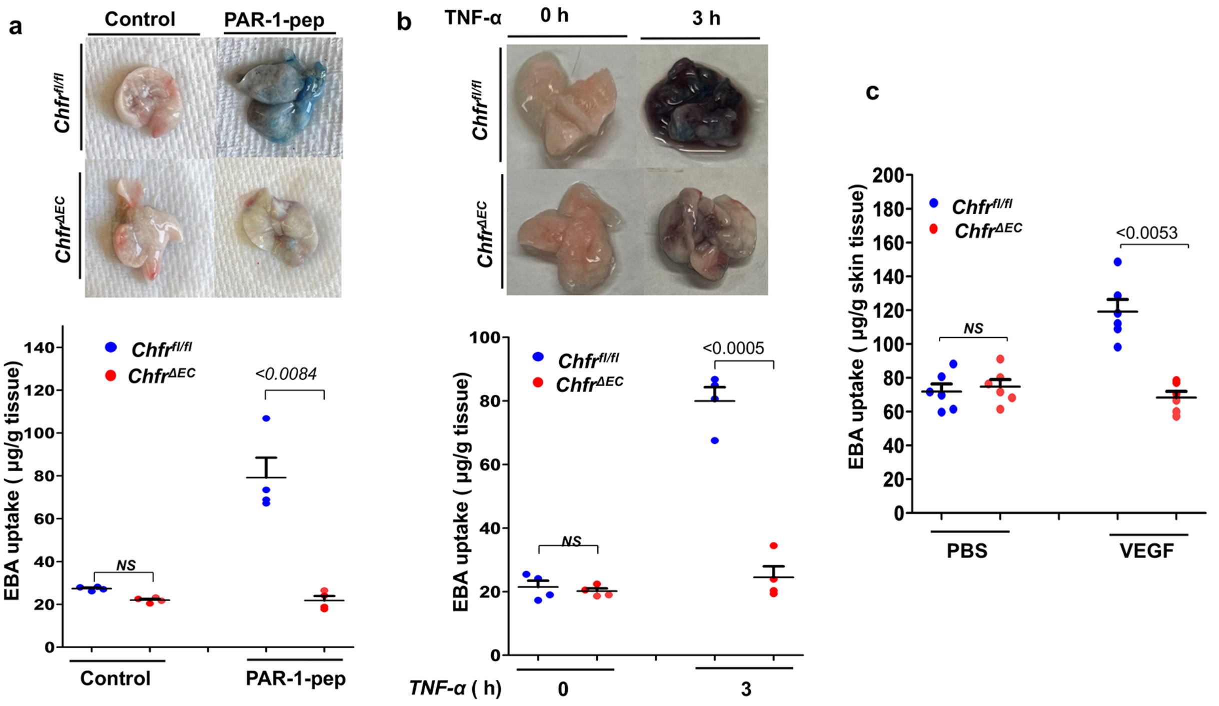

Supplementary Fig. 3. ***Chfr*** **deficiency in endothelial cells of mice blocks PAR-1-, TNF-α-, or VEGF-induced vascular permeability.** **a,** PAR-1-activating peptide-induced lung vascular leak was measured in *Chfr*$^{fl/fl}$ and *Chfr*$^{\Delta EC}$ mice. *Chfr*$^{fl/fl}$ and *Chfr*$^{\Delta EC}$ mice received PAR-1-activating peptide (TFLLRNPNDK-NH_2; 1 mg/mouse) intravenously (i.v.) and after 15 min, mice were i.v. injected with Evans blue (EB) dye (70 µg/g body weight). Thirty minutes after EB dye injection, lungs harvested were used to measure EB dye content in the lung tissue. PAR-1-pep, PAR-1-activating peptide; Shown are mean values ± SEM (n = 4 mice/genotype; unpaired two-tailed Student's *t*-test). **b**, TNF-α-induced lung vascular leak was measured in *Chfr*$^{fl/fl}$ and *Chfr*$^{\Delta EC}$ mice. *Chfr*$^{fl/fl}$ and *Chfr*$^{\Delta EC}$ mice received recombinant TNF-α (100,000 units/mouse) i.v. and after 3 h, mice were i.v. injected with EB dye as above to measure dye content in the lung tissue. Shown are mean values ± SEM (n = 4 mice/genotype; unpaired two-tailed Student's *t*-test). **c**, VEGF-induced vascular leak in the skin was measured in *Chfr*$^{fl/fl}$ and *Chfr*$^{\Delta EC}$ mice. Mice were first injected EB dye (70 µg/g body weight) and 10 min after dye injection, 20 µl of PBS or 20 µl VEGF in PBS (100 ng/site) injected intradermally into the back side of the shaved skin. Twenty min later, injected skin area excised for EB dye content analysis. Shown are mean values ± SEM (n = 4 mice/genotype; unpaired two-tailed Student's *t*-test).

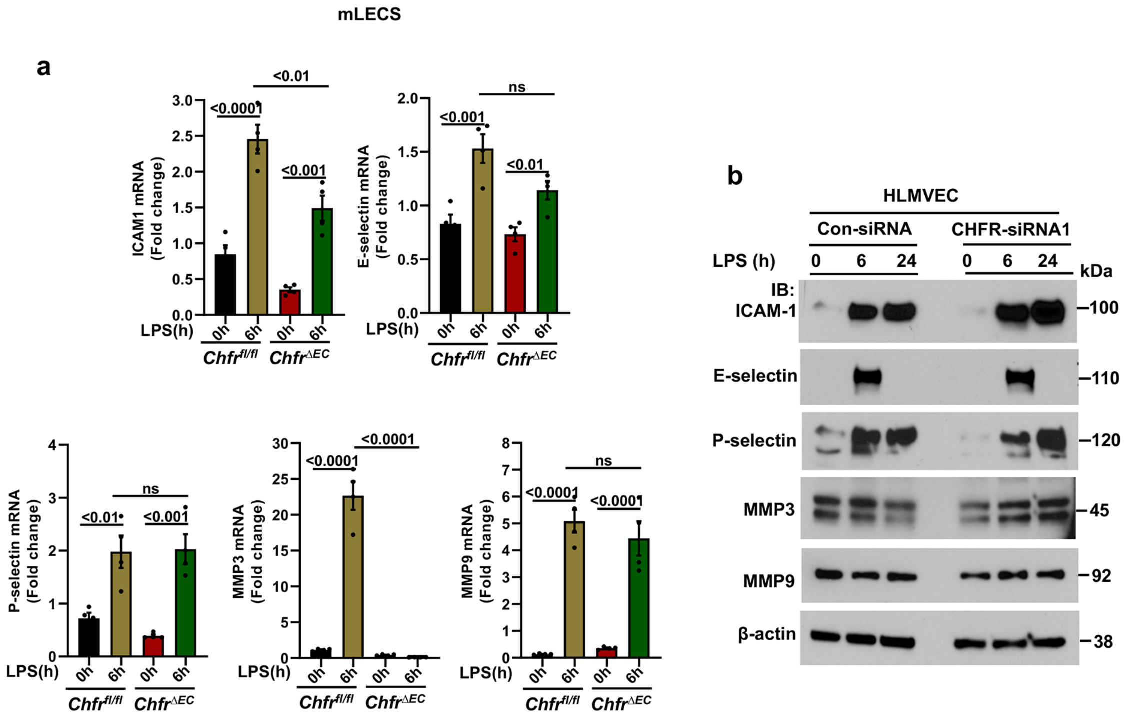

Supplementary Fig. 4. Endothelial CHFR mitigates TLR4-induced expression of ICAM-1, E-selectin, P-selectin, MMP3, and MMP9. $Chfr^{fl/fl}$ (WT) and $Chfr^{\Delta EC}$ mice were injected i.p. with LPS (10 mg/kg body weight) for 0 and 6 h. After LPS challenge, lung endothelial cells (LECs) isolated were used for total mRNA preparation and then RT-qPCR was performed. Shown are mean values $\pm$ SEM. ns, not significant (n = 4 mice/genotype/group; one-way ANOVA followed by Tukey's Post hoc test). **b**, HLMVEC were transfected with 100 nM of either scrambled-siRNA (sc-siRNA) or CHFR-siRNA1. At 48 h after transfection, cells were challenged with LPS (5 μg/ml) for 0, 6, and 24 h and then cell lysates were used for IB analysis. Results show a representative blot (n =2 independent experiments).

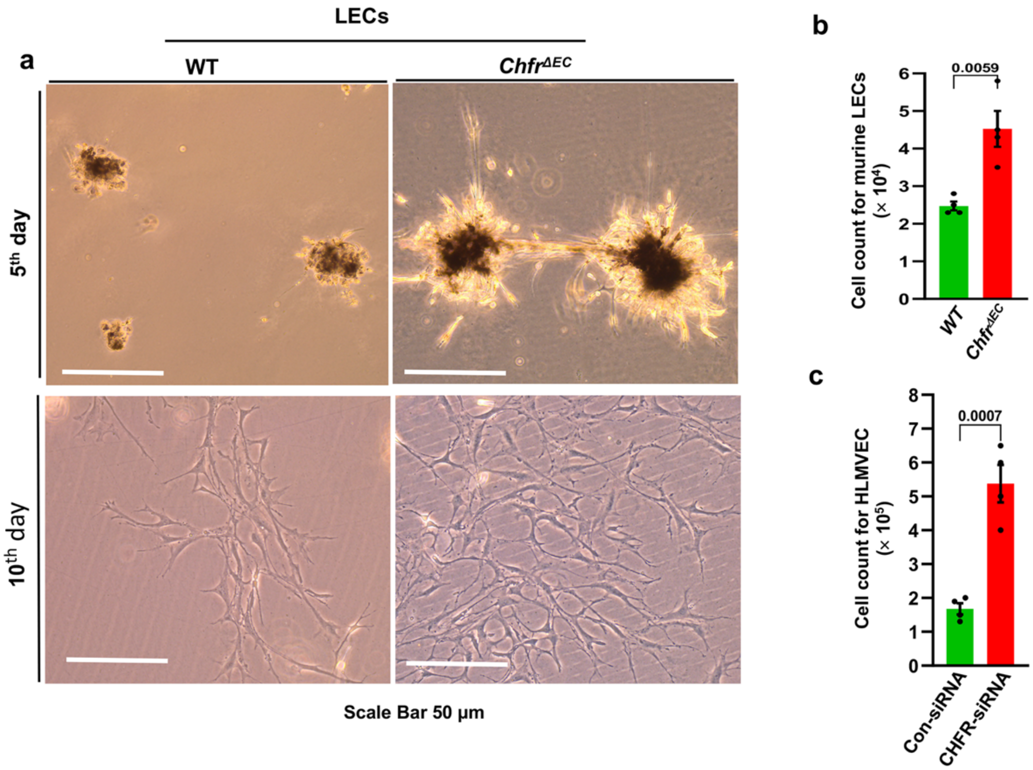

Supplementary Fig. 5. CHFR deficiency augments endothelial cell proliferation. **a**, Lung endothelial cells (LECs) from *Chfr*$^{fl/fl}$ (WT) and *Chfr*$^{\Delta EC}$ mice were placed on a Matrigel (Corning catalog #REF354234) coated 12 well plate (20,000 cell/well). Bright-field microscopic images show the endothelial cell sprouting and tube formation were increased in *Chfr*$^{\Delta EC}$ mice compared with WT mice (n = 3 independent experiments). **b**, Lung endothelial cells (LECs) from *Chfr*$^{fl/fl}$ (WT) and *Chfr*$^{\Delta EC}$ mice were seeded on 35 mm culture dishes (20,000 cell/dish). At 6th day after seeding, cells released by trypsin treatment were counted. Shown are mean values ± SEM (n = 4 dishes/genotype; unpaired two-tailed Student's *t*-test). **c**, HLMVEC were transfected with 100 nM of either scrambled-siRNA (sc-siRNA) or CHFR-siRNA1. At 48 h after transfection, cells were seeded on 35 mm culture dishes (60,000 cells/dish). At 5th day, cells released by trypsin treatment were counted. Shown are mean values ± SEM (n = 4 dishes/group; unpaired two-tailed Student's *t*-test).
