# Supplementary figures and images for: Ubiquitin ligase CHFR mediated degradation of VE-cadherin through ubiquitylation disrupts endothelial adherens junctions
Source: Nat Commun. 2023 Oct 18;14:6582. doi: 10.1038/s41467-023-42225-2 (PMC10584835; doi:10.1038/s41467-023-42225-2)

Figure 1

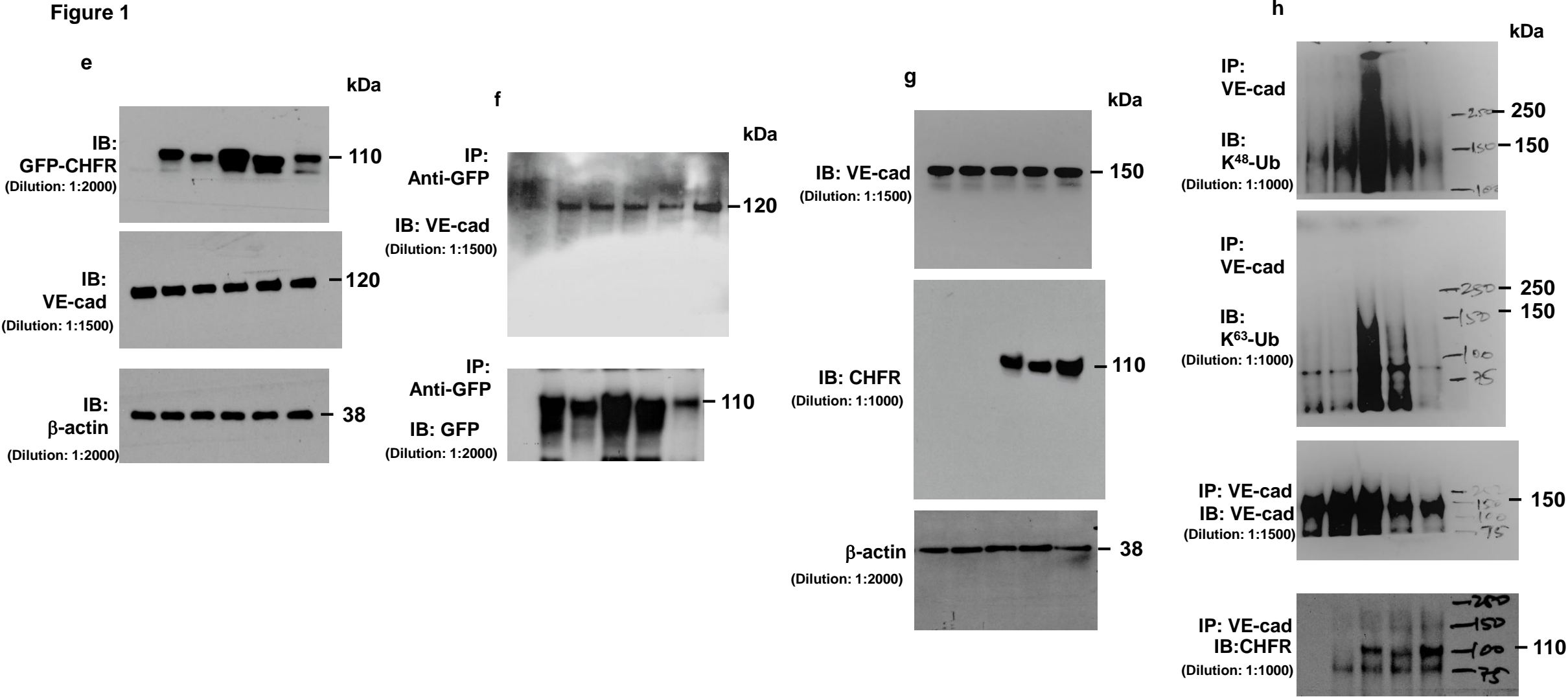

Figure 2

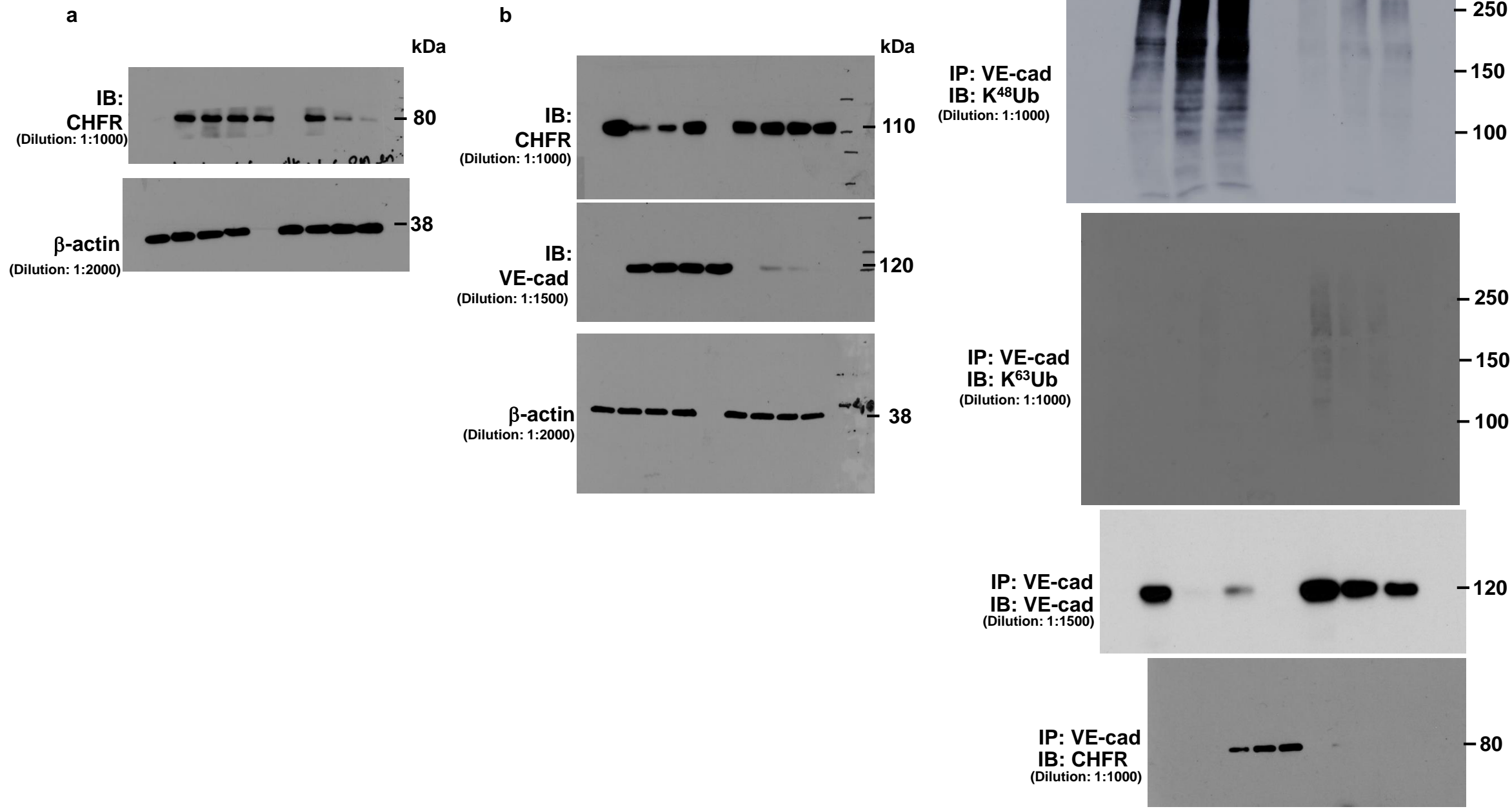

Figure 3

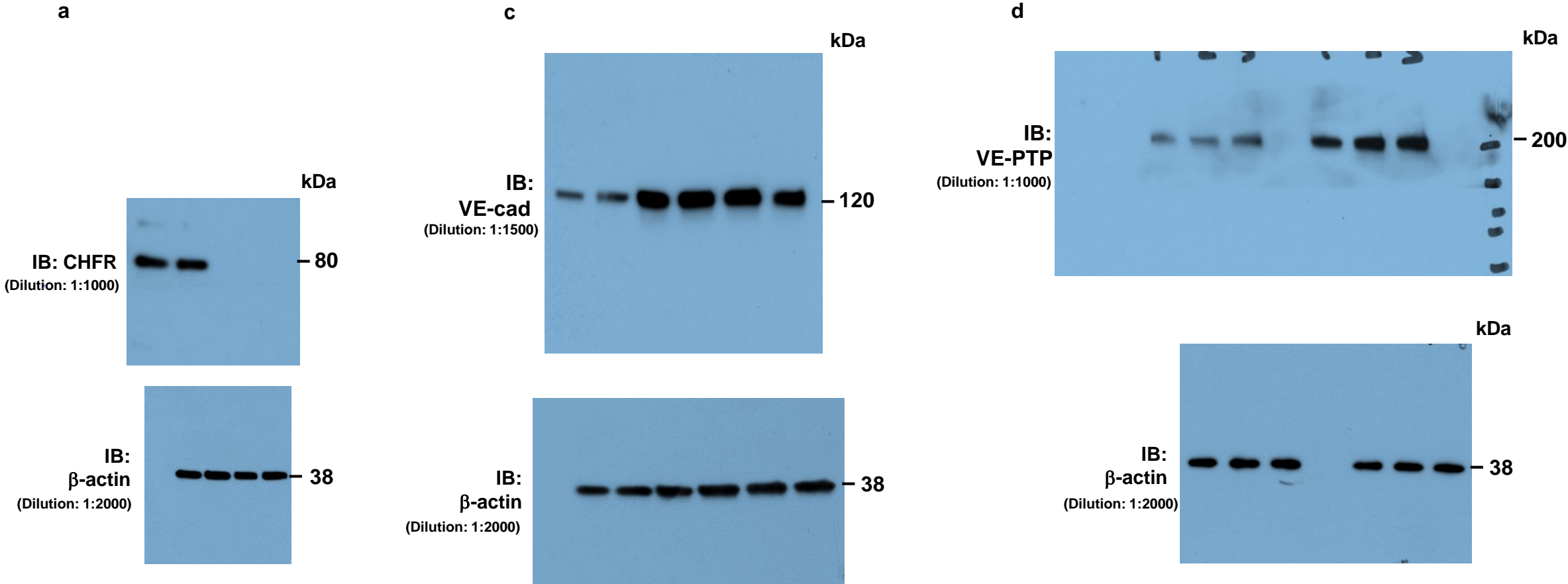

**Figure 3**

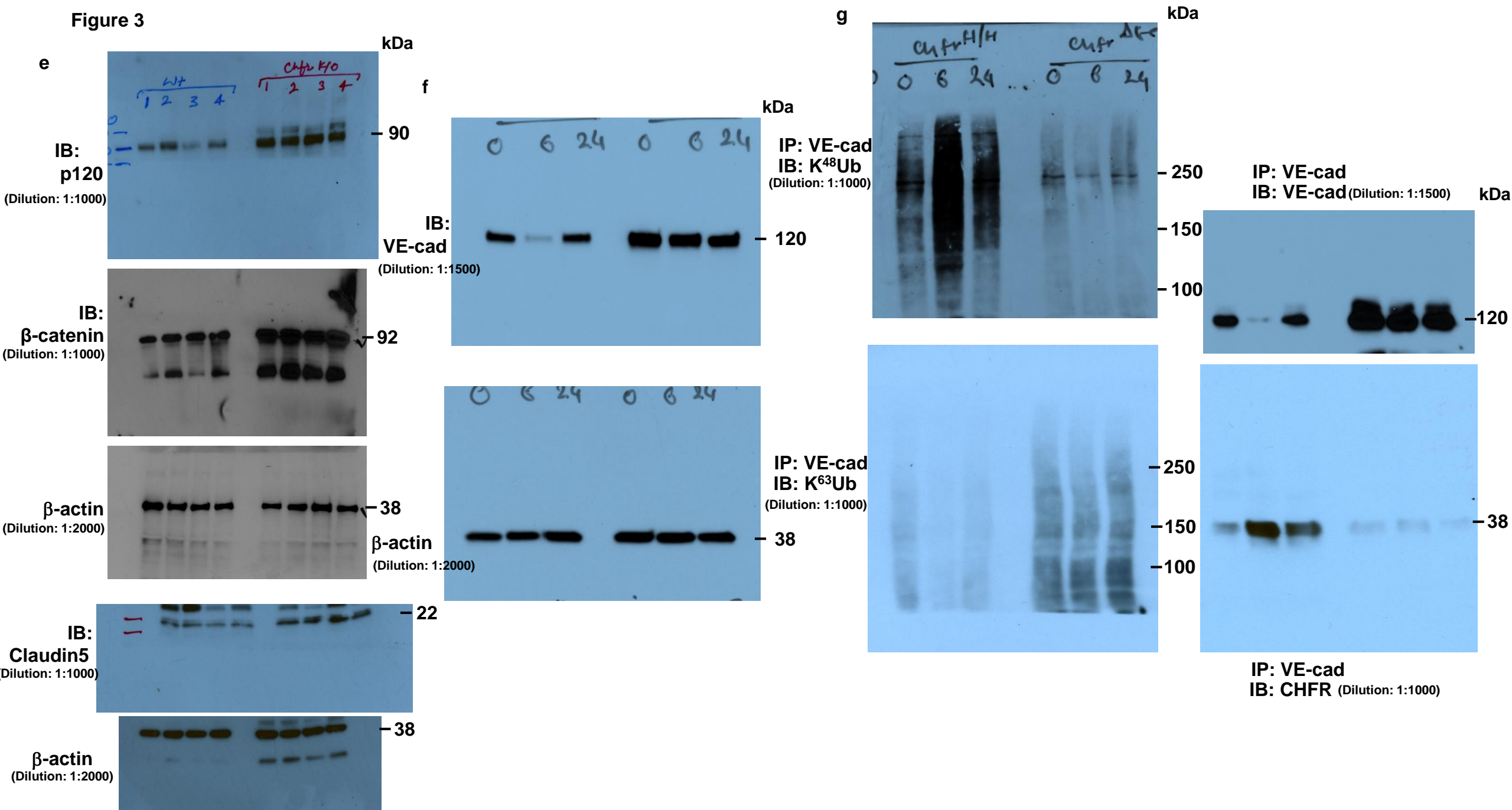

Figure 6

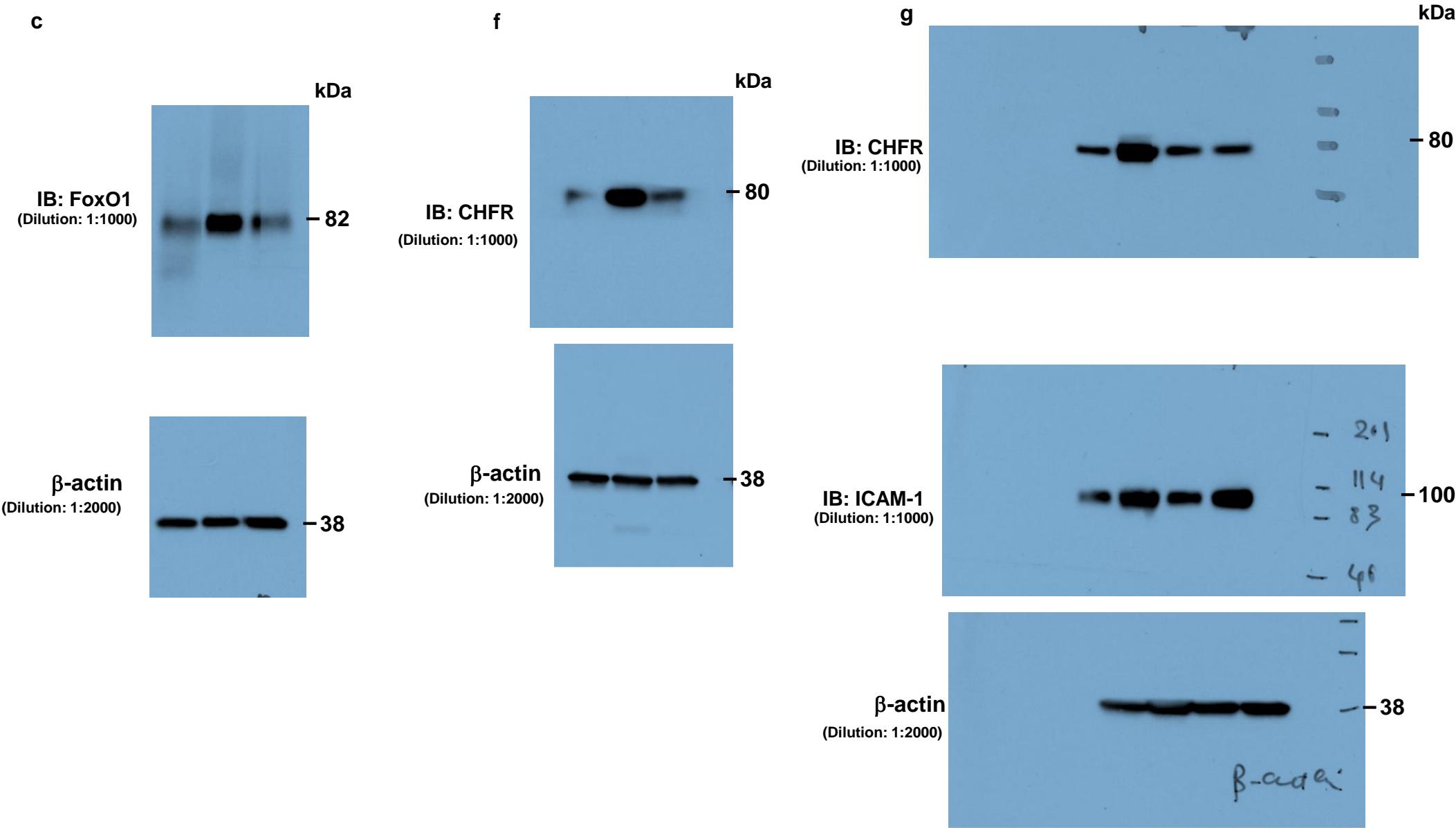

Figure 7

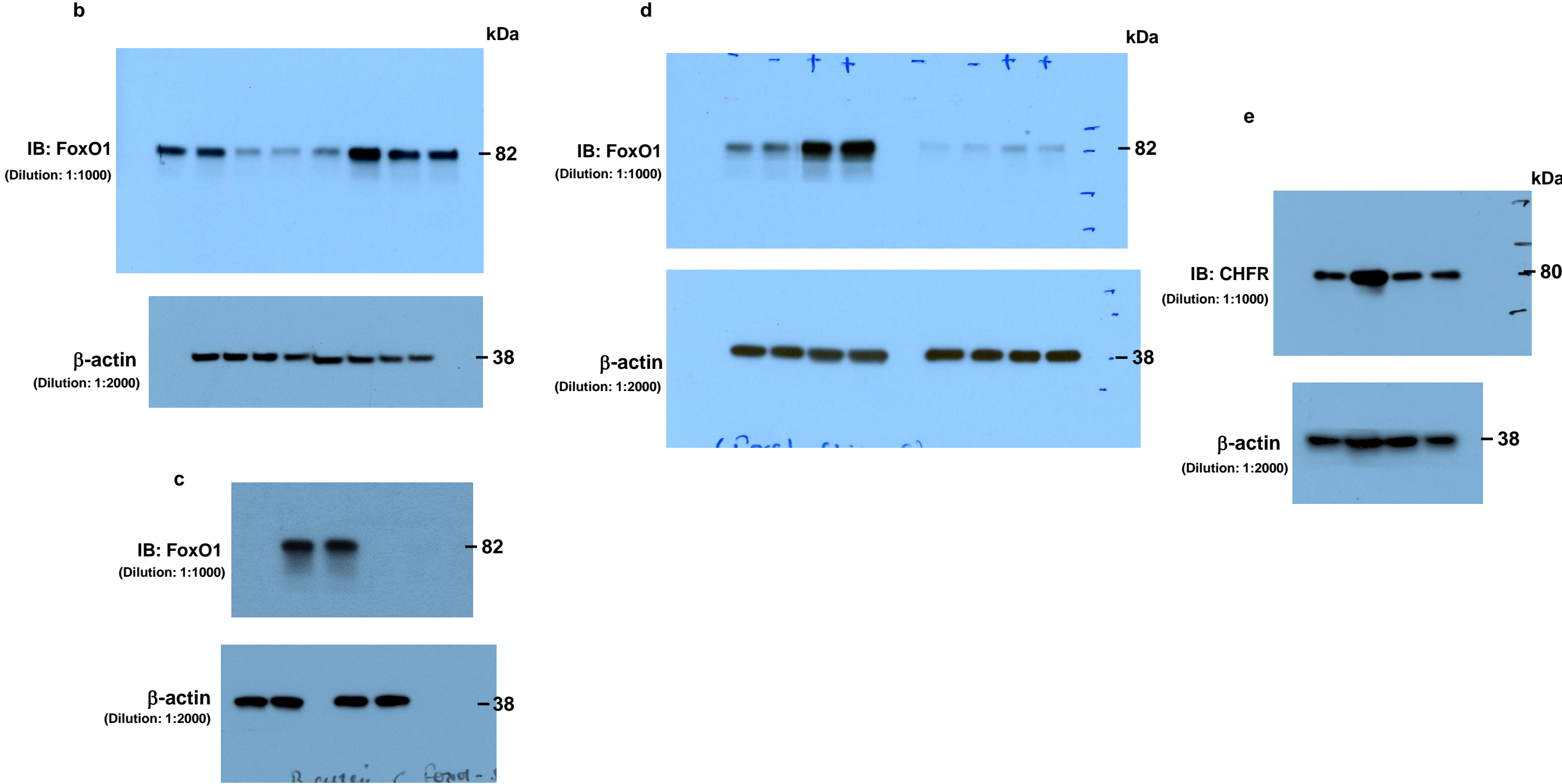

Figure 7

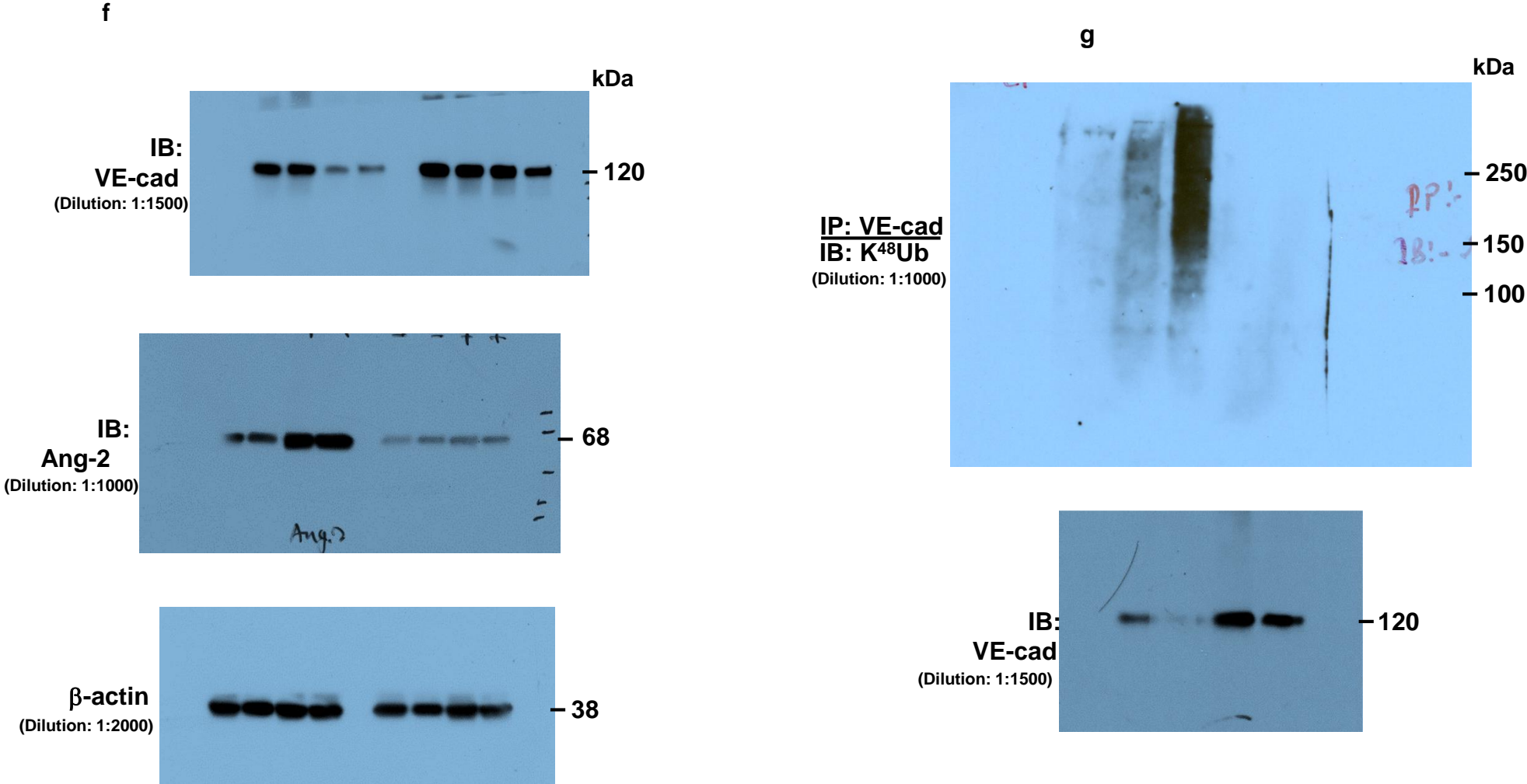

a

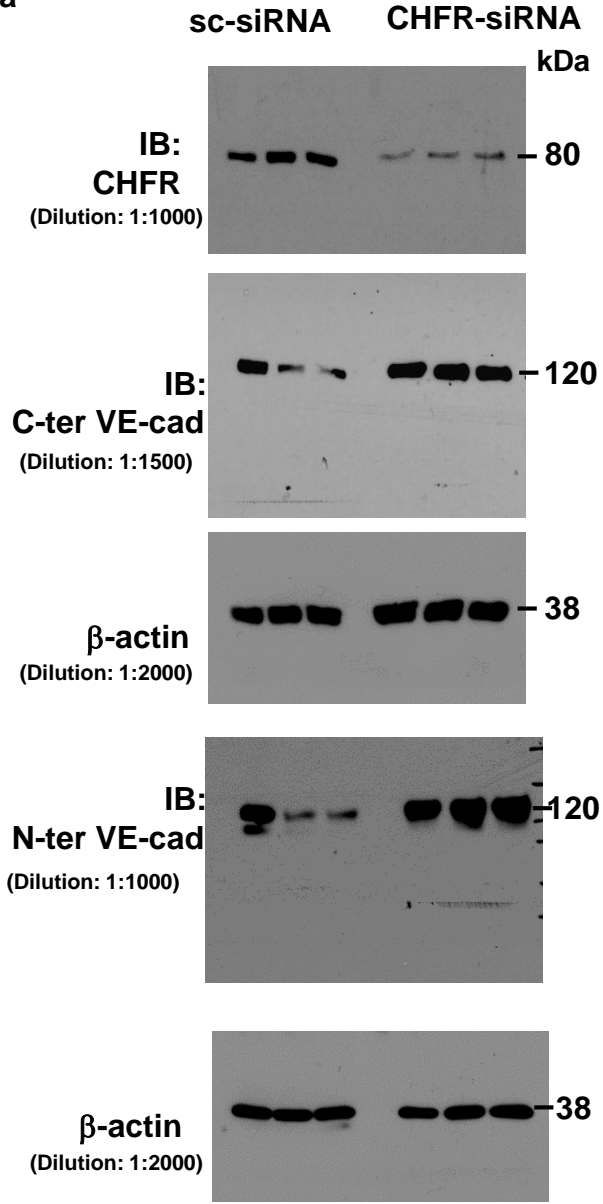

b

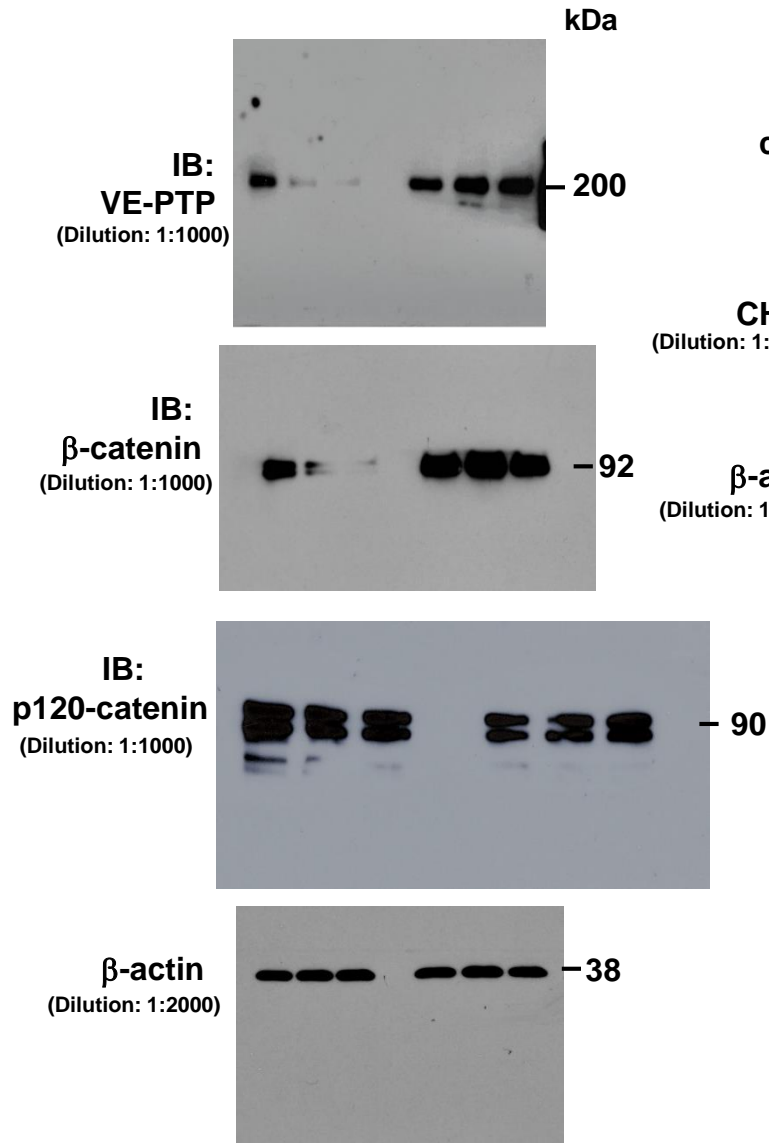

c

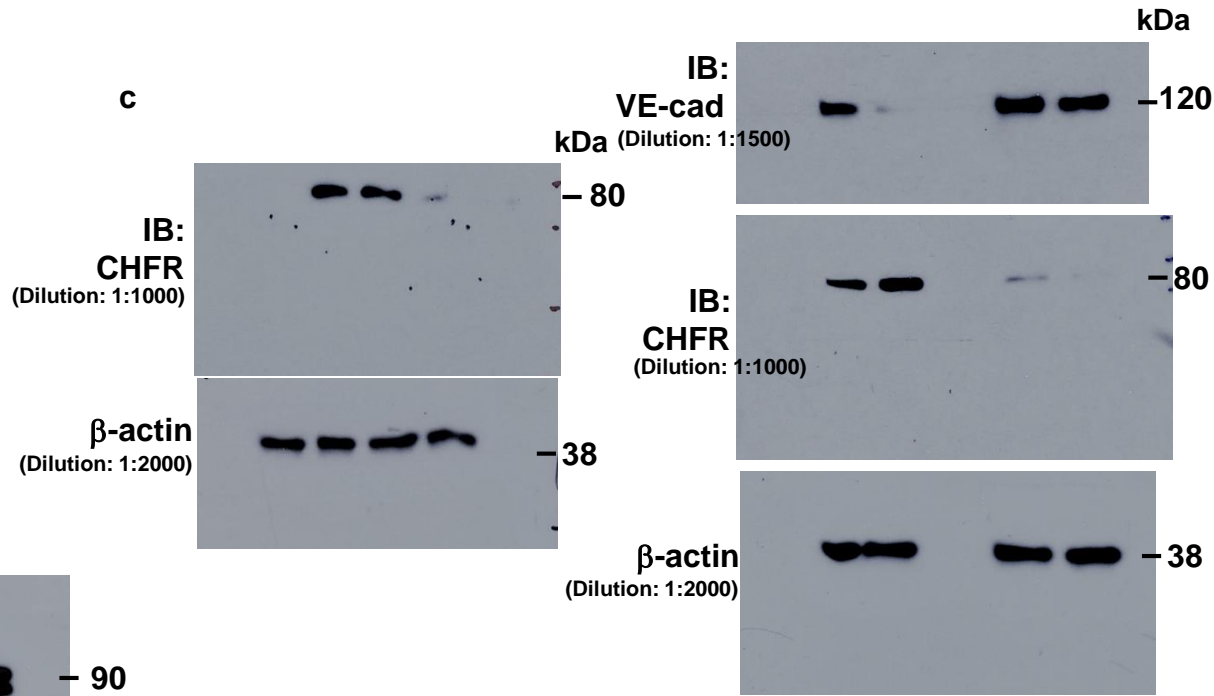

Supplementary  
Figure 4

b

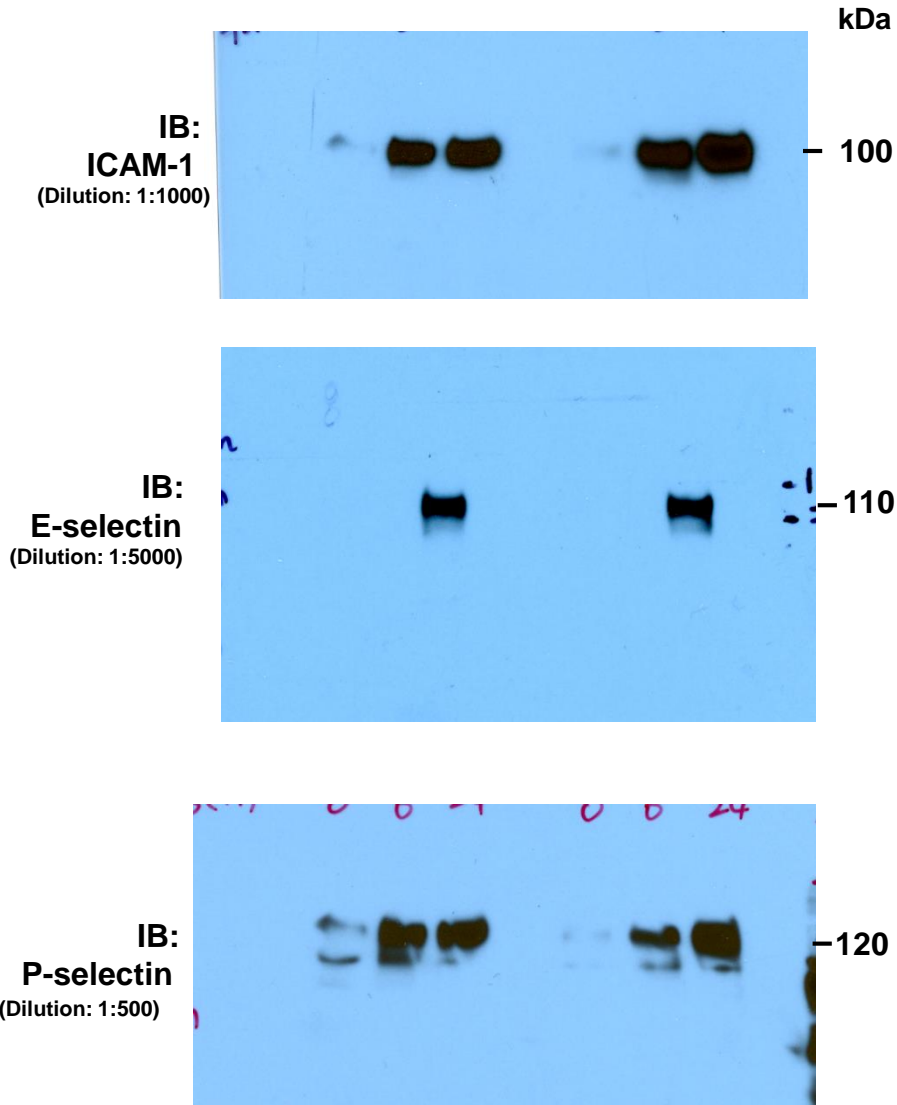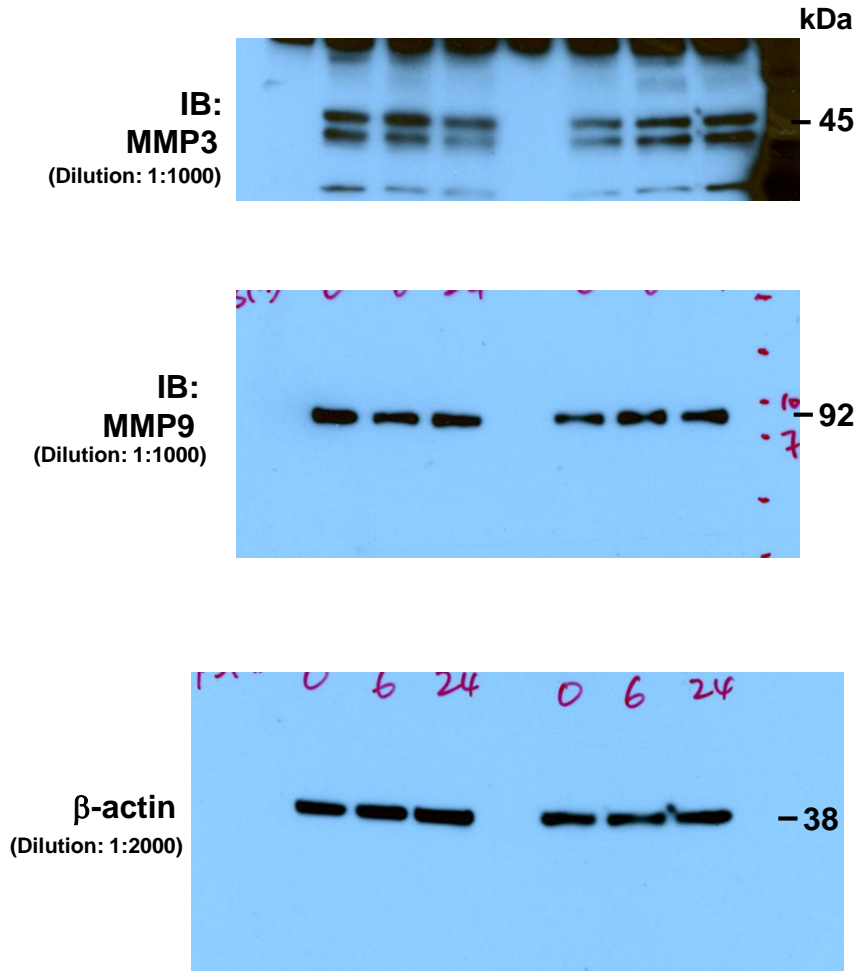

Supplement: Supplementary file 3 — Source Data [file 41467_2023_42225_MOESM3_ESM.zip › NCOMMS 22-37664C-source data file-09-29-2023/NCOMMS-22-37664C-uncropped WB-09-29-2023.pdf]
